# Whole Genome Sequence of Multiple Myeloma-Prone C57BL/KaLwRij Mouse Strain Suggests the Origin of Disease Involves Multiple Cell Types

**DOI:** 10.1371/journal.pone.0127828

**Published:** 2015-05-28

**Authors:** Sarah R. Amend, William C. Wilson, Liang Chu, Lan Lu, Pengyuan Liu, Daniel Serie, Xinming Su, Yalin Xu, Dingyan Wang, Anthony Gramolini, Xiao-Yan Wen, Julie O’Neal, Michelle Hurchla, Celine M. Vachon, Graham Colditz, Ravi Vij, Katherine N. Weilbaecher, Michael H. Tomasson

**Affiliations:** 1 Division of Oncology, Washington University School of Medicine, St. Louis, MO, United States of America; 2 Medical College of Wisconsin, Milwaukee, WI, United States of America; 3 Department of Surgery, Washington University School of Medicine, St. Louis, MO, United States of America; 4 Department of Physiology, University of Toronto, Toronto, Canada; 5 Department of Health Sciences Research, Division of Epidemiology, Mayo Clinic College of Medicine, Rochester, MN, United States of America; University of Oxford, UNITED KINGDOM

## Abstract

Monoclonal gammopathy of undetermined significance (MGUS) is the requisite precursor to multiple myeloma (MM), a malignancy of antibody-producing plasma B-cells. The genetic basis of MGUS and its progression to MM remains poorly understood. C57BL/KaLwRij (KaLwRij) is a spontaneously-derived inbred mouse strain with a high frequency of benign idiopathic paraproteinemia (BIP), a phenotype with similarities to MGUS including progression to MM. Using mouse haplotype analysis, human MM SNP array data, and whole exome and whole genome sequencing of KaLwRij mice, we identified novel KaLwRij gene variants, including deletion of *Samsn1* and deleterious point mutations in *Tnfrsf22* and *Tnfrsf23*. These variants significantly affected multiple cell types implicated in MM pathogenesis including B-cells, macrophages, and bone marrow stromal cells. These data demonstrate that multiple cell types contribute to MM development prior to the acquisition of somatic driver mutations in KaLwRij mice, and suggest that MM may an inherently non-cell autonomous malignancy.

## Introduction

Multiple myeloma (MM) is the second most common hematological malignancy and is characterized by accumulation of clonal plasma B-cells in the bone marrow, hypercalcemia, renal failure, anemia, and lytic bone lesions. Despite impressive recent progress in treatments for MM, median survival is only 6 years [1]. The requisite precursor to MM is monoclonal gammopathy of undetermined significance (MGUS), a pre-neoplastic proliferation of clonally derived plasma cells without end organ damage.

Epidemiological studies suggest a genetic component to MM disease risk that is due to an increase MGUS development. African Americans have an increased risk of MM due to elevated MGUS risk, rather than an increased rate of conversion from MGUS to overt MM [2–4]. Moreover, inherited risk variants for MM also confer a significant increase in risk for MGUS [5], providing further evidence that increased MM susceptibility is due to inherited predisposition to MGUS. Interestingly, the somatic mutations responsible for disease progression from MGUS to MM remain unknown. The majority of somatic mutations found in CD138-selected MM cells are present at similar frequencies in similar cells isolated from MGUS patients [6], suggesting that plasma-cell extrinsic factors may contribute to disease progression in MM.

Few experimental models exist to study the biology of MGUS and MM in a laboratory setting, confounding efforts to understand the biology of MM progression. Xenograft models to study human myeloma growth, e.g. SCID-hu, require the presence of a human bone marrow microenvironment [7], highlighting the importance of non-malignant cells within the myeloma microenvironment.

The C57BL/KaLwRij mouse (KaLwRij) is a spontaneously-derived inbred mouse strain identified nearly 40 years ago that is predisposed to myeloma [8]. KaLwRij mice develop benign idiopathic paraproteinemia (BIP), a condition analogous to human MGUS, at a high rate. Affected mice progress to myeloma at the same low rate as humans do, approximately 1% per year. The 5TGM1 cell line originally isolated from a sick KaLwRij mouse is often used as a model of myeloma because when transplanted back into KaLwRij mice, it causes disease with similar clinical features as human MM including lytic bone lesions [8]. Notably, while the 5TGM1/KaLwRij model recapitulates many features of human disease, the genetic basis of BIP susceptibility in KaLwRij mice remains unknown.

In this study, we investigated the genetic determinants underlying the BIP predisposition of the KaLwRij strain. Using an integrative genetics approach, including whole genome and exome sequencing, we identified novel KaLwRij gene variants, such as homozygous deletion of *Samsn1* and deleterious point mutations in tumor necrosis factor receptor family members. KaLwRij genetic variants significantly affected multiple cell types implicated in MM pathogenesis including B-cells, macrophages, and bone marrow stromal cells. These results illuminate pathways responsible for MM disease risk, and demonstrate for the first time that the development of myeloma involves multiple cell types prior to the acquisition of somatic mutations.

## Results

**Fig 1:**
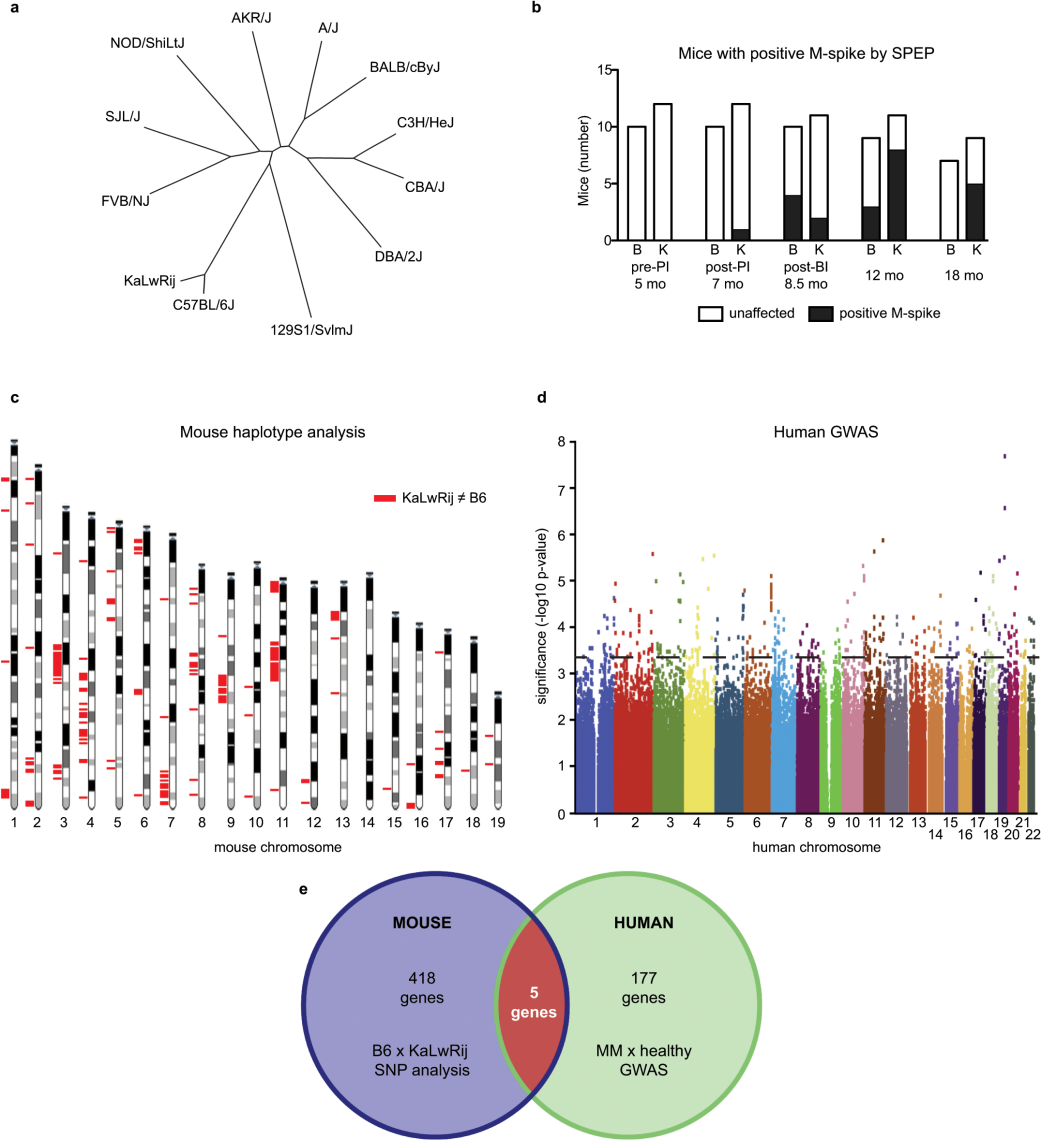
The KaLwRij strain was predisposed to BIP and intersecting mouse and human genetic analyses identified candidate genes that may influence murine BIP risk and human MM risk. (a) Phylogenetic tree demonstrating genetic distances of 12 inbred strains of mice. (b) Number of C57BL/6 and KaLwRij mice with positive M-spike by SPEP. (c) Haplotype analysis identified contiguous regions of non-shared polymorphic alleles between KaLwRij and C57BL/6 mice (red bars) in 419 genes. (D) GWAS between MM patients and healthy volunteers. SNPs in the 99th percentile (dashed line) fell in 178 genes. (e) Venn diagram representing combined analysis of (c) and (d), resulting in a candidate gene list of 5 genes.

We mapped genetic distances among myeloma-prone KaLwRij and eleven diverse inbred mouse strains using SNP arrays. KaLwRij was most closely related to its parent strain C57BL/6 (Fig 1A). Initially we hypothesized that KaLwRij predisposition to BIP would be reflected in a unique antibody response to immune challenge and that sustained serum immunoglobulin levels would provide a measurable quantitative phenotype to perform quantitative trait loci (QTL) mapping. Following immunization of these twelve strains (S1A Fig), analysis of serial serum samples by immunoglobulin ELISA demonstrated that the antibody response was highly heritable (IgG h^2^ = 0.7247, IgM h^2^ = 0.9551, IgA h^2^ = 1.019), indicating influence by genetic background (S1B–S1D Fig). Serum protein electrophoresis (SPEP), a standard diagnostic test for human MGUS, was used to identify M-spikes indicative of BIP (S1E Fig). Most strains presented with an M-spike immediately following immunization, indicating a normal immune response (S1 Table). M-spike presentation may be due either to increased survival of plasma cells or increased activation of memory B-cells, but work beyond the scope of this manuscript is necessary to dissect these possibilities. The highest frequency of an abnormal M-spike sustained to 18 months was found in KaLwRij (56%) while it had resolved in C57BL/6 mice (Fig 1B). The 18-month time frame and qualitative nature of the BIP phenotype prevented us from further pursuing QTL mapping.

We took advantage of the close genetic distance between BIP-resistant C57BL/6 and BIP-susceptible KaLwRij mouse strains to use haplotype mapping to identify BIP candidate genes. Of 562,061 single nucleotide polymorphisms (SNPs) queried, 21,133 SNPs varied between KaLwRij and C57BL/6 (3.76%). A ranked list, defined by blocks of five or greater physically consecutive divergent SNPs, identified 418 candidate genes different between C57BL/6 and KaLwRij (Fig 1C, S2 Table). To enrich for candidate genes relevant to human MM, we took an integrative cross-species approach. We performed genome-wide association analysis (GWAS) on genomic DNA isolated from normal tissue of 305 MM patients and 353 healthy controls to identify common genetic variants associated with MM. The relatively small patient population identified only one SNP (rs1029654 in an intergenic region) that reached genome-wide significance. To include additional genetic variants associated with MM risk, we queried SNPs in the 99th significance percentile (209 SNPs, Fig 1D) and generated a candidate gene list of 177 genes possibly influencing MM risk in humans (S3 Table). Importantly, this approach identified SNPs in three of the seven previously published genetic loci associated with MGUS and MM risk (2p23.3, 3p22.1, and 7p15.3), validating our approach. The intersection of the KaLwRij and C57BL/6 haplotype gene set (418 genes) and the human GWAS set (177 genes) contained five genes: *Fstl4*, *Samsn1*, *Ccm2*, *Tenm3*, and *Csmd1* (Fig 1E).

To characterize these loci at base-pair resolution and to identify additional genomic variants contributing to MM pathogenesis, we performed whole genome sequencing (WGS) and whole exome sequencing (WES). 926,326,580 reads were obtained by WGS and 75,950,592 by WES, with 96.0% and 98.9% mapping to the reference C57BL/6 genome respectively. These data were analyzed for large deletions, single nucleotide variants (SNVs), and small insertion or deletion events (S4–S7 Tables). 19,042 cross-validated SNVs were identified in the KaLwRij genome (S5 Table and data not shown). Of these SNVs, 1,128 (5.9%) resulted in non-synonymous coding sequence changes (S5 Table), with 29 novel variants predicted to be disruptive (Table 1).

**Table 1:** KaLwRij novel germline missense, stoploss, and stopgain mutations.

Chr	Position (bp)	Gene ID	Gene Name	Variant
2	20861102	*Arhgap21*	Rho GTPase activating protein 21	c.C3215T:p.T1072I
3	95130195	*Scnm1*	Sodium channel modifier 1	c.T559C:p.stop187R
4	88403392	*Focad*	Focadhesin	c.A4979T:p.N1660I
7	140274118	*5830411N06Rik*	Riken cDNA 5830411N06 gene	c.C1033T:p.Q345stop
7	140466879	*Olfr533*	Olfactory receptor protein 533	c.G677A:p.R226H
7	140466884	*Olfr533*	Olfactory receptor protein 533	c.C682T:p.R228C
7	140503539	*Olfr536*	Olfactory receptor protein 536	c.C919T:p.R307C
7	140691588	*Olfr45*	Olfactory receptor protein 45	c.C682T:p.R228C
7	143643365	*Tnfrsf22*	Tumor necrosis factor receptor superfamily, member 22	c.A236C:p.Q79P
7	143680034	*Tnfrsf23*	Tumor necrosis factor receptor superfamily, member 23	c.A206C:p.Q69P
8	53513574	*Aga*	Aspartylglucosaminidase	c.C160A:p.L54M
11	3179430	*Sfi1*	Sfi1 homolog, spindle assembly associated (yeast)	c.G344A:p.W115stop
11	3923487	*Tcn2*	Transcobalamin 2	c.C858G:p.F286L
11	4075493	*Sec14l3*	SEC14-like 3 (S. cerevisiae)	c.C1016A:p.T339N
11	5144386	*Emid1*	EMI domain containing 1	c.A124G:p.T42A
11	43490831	*C1qtnf2*	C1q and tumor necrosis factor related protein 2	c.G379A:p.G127R
11	49294204	*Olfr1392*	Olfactory receptor protein 1392	c.A882T:p.K294N
11	50833599	*Zfp879*	Zinc finger protein 879	c.G410T:p.G137V
11	51007734	*Olfr51*	Olfactory receptor protein 51	c.C761T:p.A254V
11	51027442	*Olfr54*	Olfactory receptor protein 54	c.G439T:p.A147S
11	52144841	*Olfr1373*	Olfactory receptor protein 1373	c.G688A:p.V230M
11	107179035	*Nol11*	Nucleolar protein 11	c.C988T:p.P330S
13	21423407	*Pgbd1*	PiggyBac transposable element derived 1	c.C505T:p.Q169stop
16	32753901	*Muc4*	Mucin 4, cell surface associated	c.C3776A:p.T1259N
17	46555608	*Srf*	Serum response factor	c.G221T:p.G74V
17	73535535	*Galnt14*	UDP-N-acetyl-alpha-D-galactosamine:polypeptide N-acetylgalactosaminyltransferase 14	c.C519A:p.N173K
18	67402164	*Tubb6*	Tubulin, beta 6 class	c.T1132G:p.F378V
X	20853392	*Araf*	v-raf murine sarcoma 3611 viral oncogene homolog	c.G632A:p.R211H
X	51130161	*Mbnl3*	Muscleblind-like 3 (Drosophila)	c.C715A:p.Q239K

**Fig 2:**
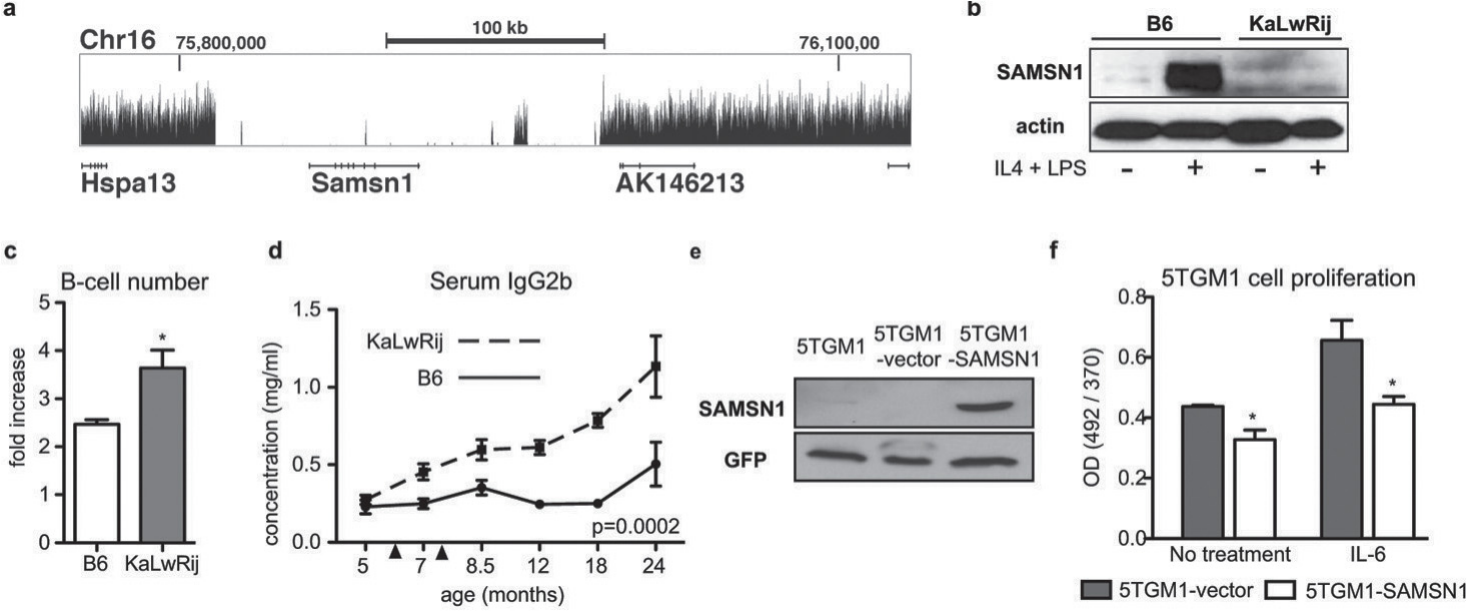
*Samsn1* is deleted in KaLwRij and was a negative regulator in B-cells and transformed myeloma cells. (a) Genome sequence coverage is shown for the *Samsn1* locus. Physical distance shown along the x-axis and the size and location of the region are indicated. Height of the curve represents accumulated sequencing reads. There are virtually no reads across *Samsn1* indicating a total deletion of this gene. *Hspa13* gene 5’ of *Samsn1* (shown and labeled) is not affected, and neither are 3’ genes AK146213 (shown and labeled) or 4930578N18Rik (first two exons shown but not labeled). *Samsn1* is encoded in reverse orientation. (b) Western blot analysis of SAMSN1 in CD43- splenic B-cells from C57BL/6 or KaLwRij mice stimulated with IL-4 and LPS for 72 hours. (c) CD43- splenic B-cells were stimulated with IL-4 and LPS for 72 hours and cell number counted pre- and post-stimulation to determine proliferation. (d) Mice were immunized (arrowheads indicate primary and secondary immunization) and serial serum samples collected. IgG2b levels were determined by ELISA. (e) Western blot for Samsn1 in parental 5TGM1 cells, control vector cells (5TGM1-vector), and cells overexpressing Samsn1 (5TGM1-SAMSN1). (f) 5TGM1-vector and 5TGM1-SAMSN1 cells were stimulated with IL-6 for 24 hours. Cell proliferation was measured by BrdU incorporation.

Analysis of structural variants identified two deletions. The first, confirmed by PCR and sequence analysis, spanned 1.6 kb within *Rfpl3s* (data not shown). More compelling, however, was a large 180 kb deletion encompassing the entire *Samsn1* gene (Chr16: 75816190–75996162; Fig 2A, S2A Fig), which was also identified in our cross-species approach.

To characterize the functional effect of *Samsn1* deletion, we utilized a genetic mouse model with targeted deletion of *Samsn1* on the C57BL/6 background. *Samsn1* ^*-/-*^ mice have increased B-cell proliferation in vitro and increased immunoglobulin response in the weeks following immunization in vivo [9]. The 180 kb deletion resulted in an effective *Samsn1* knock-out in KaLwRij mice, so we predicted KaLwRij mice would display similar B-cell phenotypes observed in *Samsn1* ^*-/-*^ mice. Samsn1 was expressed in C57BL/6 but not in KaLwRij ex vivo stimulated splenic B-cells, confirming the loss of protein expression predicted by gene deletion (Fig 2B). B-cells isolated from young KaLwRij prior to onset of BIP and myeloma had significantly increased proliferation following stimulation compared to C57BL/6 (Fig 2C). Additionally, KaLwRij mice had a significant and progressive elevation in immunoglobulin IgG2b levels following immunization (Fig 2D).

We next tested whether *Samsn1* expression played a role in fully transformed myeloma cells. The 5TGM1 myeloma cell line, originally isolated from a myeloma-bearing KaLwRij mouse [10], was confirmed as *Samsn1*-null (Fig 2E, S2A Fig). Stable re-expression of *Samsn1* decreased proliferation of 5TGM1 cells compared to control under both basal and IL-6 stimulated conditions (Fig 2F). Taken together, these data demonstrate that *Samsn1* regulated B-cell proliferation in non-tumor bearing mice and restrained MM tumor cell growth in part through a plasma cell intrinsic mechanism.

*SAMSN1* (*HACS1*) was first cloned on the basis of its differential expression in multiple myeloma with low expression in human myeloma cell lines [11]. To further investigate whether *SAMSN1* participates in human myeloma, we queried plasma-cell *SAMSN1* gene expression in patient samples. *SAMSN1* was expressed at lower levels in both human MGUS and MM cells compared to normal human plasma cells (S3 Fig), suggesting that plasma-cell intrinsic *SAMSN1* may also play a role in human MM progression.

**Fig 3:**
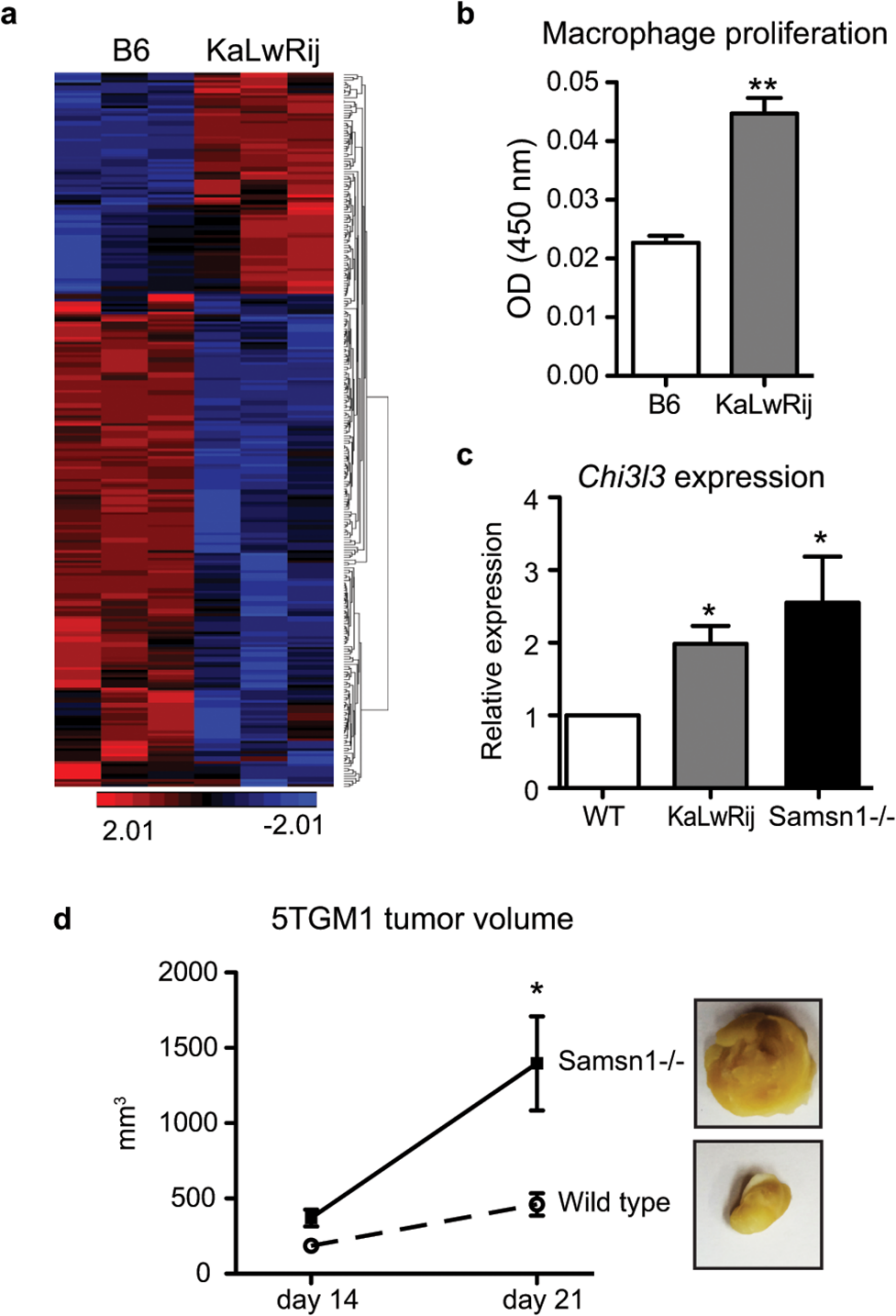
Loss of Samsn1 enhanced pro-tumor macrophage function. (a) Microarray analysis of gene expression in C57BL/6 and KaLwRij primary bone marrow macrophages. (b) Proliferation of C57BL/6 and KaLwRij macrophages was measured by MTT assay. (c) Macrophage M2 polarization marker Chi3l3 in bone marrow macrophages. (d) Wild-type or *Samsn1* ^*-/-*^ M2 macrophages polarized ex vivo were injected directly into established 5TGM1 tumors and tumor burden was monitored by bidirectional caliper measurement.

*Samsn1* expression was not restricted to the B-cell lineage (S2B Fig), so we hypothesized that *Samsn1* might influence MM development via effects on additional cell types in the tumor stroma. We examined two cell types known to participate in MM pathogenesis: macrophages (*Samsn1* expressors) and bone marrow stromal cells (BMSCs, *Samsn1* non-expressors; S2B Fig). Microarray analysis of BIP-resistant C57BL/6 and BIP-susceptible KaLwRij primary bone marrow macrophages identified 281 differentially expressed genes (Fig 3A, S8 Table). KaLwRij macrophages had increased proliferation (Fig 3B) and increased *Chi3l3* (1.80 fold), *Chi3l1* (2.48 fold), and *Cxcl2* (3.45 fold) expression, transcriptional markers for pro-tumor M2 macrophage polarization. Notably, there were no variants identified in these genes in the KaLwRij genome. *Chi3l3* expression was also significantly increased in macrophages isolated from *Samsn*1^-/-^ mice, confirming that these macrophage expression changes were due specifically to *Samsn1* deficiency (Fig 3C).

To test the function of *Samsn1* in tumor-associated macrophages in vivo, we injected either wild-type or *Samsn1* ^-/-^ ex vivo polarized M2 macrophages into established 5TGM1 tumors. Compared to wild-type, *Samsn1* ^-/-^ M2 macrophages significantly increased the growth of myeloma tumors (Fig 3D). These findings indicate that *Samsn1* regulates a pro-tumor function of macrophages in the myeloma microenvironment.

**Fig 4:**
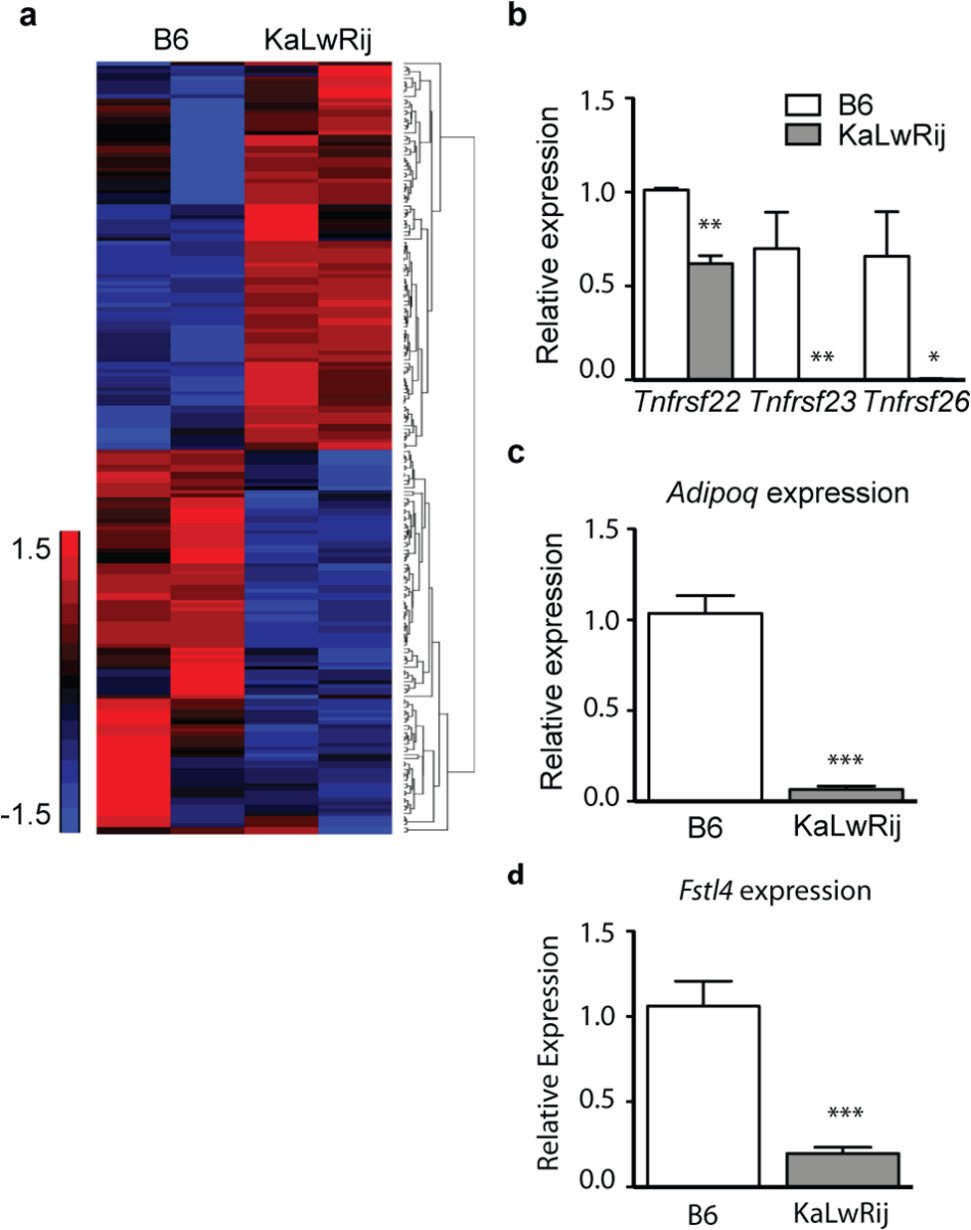
KaLwRij bone marrow stromal cells (BMSCs) have altered gene expression profiles independent of *Samsn1*. (a) Microarray analysis of B6 and KaLwRij primary BMSCs. (b-d) RT-qPCR analysis of *Tnfrsf22*, *Tnfrsf23*, *Tnfrsf26*, *Adipoq*, and *Fstl4* mRNA levels in B6 and KaLwRij BMSCs. * P >0.05, ** P > 0.005, *** P < 0.0005.

Interestingly, microarray analysis of *Samsn1*-nonexpressing BMSCs from C57Bl/6 and KaLwRij also showed many gene expression differences (Fig 4A). *Tnfrsf22*, *Tnfrsf23*, and *Tnfrsf26*, identified in the WGS (S5 Table), showed lower expression in KaLwRij BMSCs compared to C57BL/6 (Fig 4B, S8 Table). Consistent with other reports, expression of *Adipoq*, previously implicated in MM biology in mice and patients [12], was also significantly reduced in BMSCs from KaLwRij (Fig 4C). *Fstl4*, initially identified by our integrative genetics approach, was also depleted at steady-state levels in BMSCs (Fig 4D).

## Discussion

High throughput WGS analyses of human MM cell genomes have revealed striking genetic diversity but have failed to clarify the etiology of MM [13, 14]. The spontaneously-derived KaLwRij inbred mouse strain is commonly used as a model for MM but the KaLwRij genome has not previously been sequenced. Here, we present the WGS of the KaLwRij mouse as well as provide gene expression profiles for MM-supportive macrophages and BMSCs as evidence for multiple cell lineages contributing to MM prior to overt disease manifestation. Rather than being confined to the B-lineage, the presumed cell of origin of MM, our results suggest that genetic susceptibility alleles are expressed in both the pre-malignant B-cell and in supportive host microenvironment cells.

Combined analysis of loci that contribute to inherited disease risk to both MM in humans and BIP in mice identified *Samsn1* as a likely candidate to influence disease susceptibility. Surprisingly, we discovered that *Samsn1* is homozygously deleted in the KaLwRij genome [15]. KaLwRij mice have a similar phenotype to *Samsn1-/-* mice including increased proliferation of B-cells in ex vivo culture and elevated IgG levels with age [9], supporting the conclusion that absence of *Samsn1* drives KaLwRij BIP. *Samsn1* add-back inhibited proliferation in transformed MM cells suggesting that the gene can play a plasma-cell intrinsic tumor suppressor role in KaLwRij mice, but we also demonstrated that the absence of *Samsn1* in macrophages also contributes to MM progression, making the role of this pathway more complex.

Most compelling were the non-plasma cell contributions of *Samsn1* to MM. The majority of genetic alterations in MM cells are already present in MGUS plasma cells [6], suggesting that plasma cell extrinsic factors contribute to the conversion of MGUS to MM. Rather than being confined to the affected plasma cell, the genetic susceptibility alleles in KaLwRij also are expressed in supportive host microenvironment cells. *Samsn1*-null macrophages had increased M2 macrophage markers and potently increased myeloma tumor growth in vivo. These data place *Samsn1* as an inhibitor of pro-tumorigenic macrophage polarization and as a plasma-cell extrinsic regulator of MM growth.

Our findings also strongly suggest that gene loci in addition to *Samsn1* are likely to be important to MM pathogenesis in the KaLwRij model. We found significant gene expression differences between C57BL/6 and KaLwRij BMSCs, a myeloma-supportive cell type in the tumor microenvironment that does not express *Samsn1* (Fig 4). Through WGS, we identified additional KaLwRij genes that may explain differences between KaLwRij and C57BL/6 BMSCs. Notably, we identified deleterious variation and decreased expression in multiple BMSC-expressed genes, including *Tnfrsf22*, *Tnfrsf23*, and *Tnfrsf26*. These genes encode decoy receptors for the TRAIL cytokine [16]. TRAIL-OPG signaling has been implicated in MM progression in humans [17], and these KaLwRij variants may support MM via paracrine signaling.

WGS also identified a novel variant in a paralog of adiponectin (*Adipoq)*, *C1qtnf2*, predicted to be disruptive. *C1qtnf2* is a ubiquitously expressed lipokine with similar function to adiponectin. *Adipoq* has been shown to have anti-myeloma effects in KaLwRij mice and in humans [12], and we confirmed significantly lower *Adipoq* expression in KaLwRij BMSCs. Together, these results provide additional lines of evidence that multiple pathways and cell types are involved in KaLwRij MM predisposition, and validate our combined genetics approach as a method for identifying pathways involved in MM pathogenesis.

*SAMSN1* expression is decreased in MGUS and MM patient samples (S3 Fig) and has reduced expression in human MM cell lines [11], but *SAMSN1* is not the target of somatic mutation in human MM [14]. Further work to map the SAMSN1 pathway in humans is needed before we can determine the significance of *SAMSN1* to human MM. While *SAMSN1* is conserved in humans, one of its murine binding partners, PIRB is not [18]. SAMSN1 is also reported to bind cortactin, a component of integrin signaling and cell migration that is conserved in humans [19], but it is not known whether this interaction is necessary for *SAMSN1*'s role in MM. We pursued investigation of the role of *Samsn1* in KaLwRij mouse BIP susceptibility on the basis of a SNP in the human *SAMSN1* locus identified in a MM patient GWAS (Fig 2). While *SAMSN1* is not deleted in human MM, components of a SAMSN1 pathway appear likely to involved in myeloma susceptibility and/or disease progression.

Our observation that the susceptible background of KaLwRij mice involves multiple cell types helps explain two persistent dilemmas in the myeloma field: i) the marked clonal heterogeneity observed in human MM samples and ii) the inability of human MM cells to engraft in immunocompromised mice without co-transplantation of myeloid and stromal cells [7]. Drug resistance and MM cell survival is well-known to involve several stroma cell types, but the assumption in the field has been that it is the malignant MM cells that subvert normal bone marrow cells to create a pro-tumor milieu. Our findings suggest for the first time that multiple cell lineages are involved in MM pathogenesis prior to disease manifestation and independently of tumor cell somatic mutations. Further evaluation of the interaction between somatic and germline genetic events in the KaLwRij model system may provide additional insights into human MM.

## Materials and Methods

### Ethics statement

The study cohort was approved by the Human Research Protection Office at Washington University School of Medicine and at the Mayo Clinic. Informed written consent from the patients was obtained in accordance with the Declaration of Helsinki. Mice were housed in shared pathogen-free conditions according the guidelines of the Division of Comparative Medicine, Washington University School of Medicine. The Washington University Animal Studies Committee approved all experiments.

### Mice

129S1/SvImJ, A/J, AKR/J, BALB/cByJ, CBA/J, C3H/HeJ, C57BL/6, DBA/2J, FVB/NJ, NOD/ShiLtJ, SJL/J, and NOD-scid–IL2Rγ mice were purchased from The Jackson Laboratory. C57Bl/KaLwRij mice were originally obtained from Gregory Mundy at Vanderbilt University. Mice were housed in shared pathogen-free conditions according the guidelines of the Division of Comparative Medicine, Washington University School of Medicine. Mice were euthanized by asphyxiation using CO_2_ chambers. The animal ethics committee approved all experiments.

*Samsn1* ^-/-^ mice were originally obtained from Dingyan Wang [9]. *Samsn1* ^-/-^ and *Samsn1* ^+/+^ mice were housed in shared pathogen-free conditions according to the guidelines from the Animal Resource Centre of the University Health Network, Princess Margaret Hospital Animal Facility.

### Primary cell culture

Splenic B-cells were negatively selected via MACS with anti-CD43 beads (Miltenyi Biotec). Cells were cultured in RPMI 1650 media, 10% FBS, 0.00035% BME, 1% penicillin-streptomycin and stimulated with 10 ng/ml LPS and 20 ng/ml IL-4 for 72 hours. Cells were characterized by FACS (CD43- / B220+ / IgD+, S5 Fig). To generate bone marrow macrophages (BMMs), whole bone marrow was cultured in αMEM, 10% FBS, 1% penicillin-streptomycin, 50 ng/ml MCSF for 3 days. Cells were characterized by FACS (GR1- / F4/80+, S7 Fig). Proliferation was measured by standard MTT assay (Sigma-Aldrich). M2 polarized macrophages were generated by stimulating BMMs with 5 ng/ml IL-4 for 24 hours. Bone marrow stromal cells (BMSCs) were generated by plating whole bone marrow cells in ascorbic acid-free αMEM, 10% FBS, 1% penicillin-streptomycin for 7 days in 5% oxygen followed by negative selection via MACS with anti-CD45 beads (Miltenyi Biotec). Cells were characterized by FACS (CD45-, S6 Fig). Flow Cytometric Analysis was performed using FACSCALIBUR (BD Biosciences) and analyzed with FlowJo software (Tree Star). Antibodies used: APC-CD45 (BD Pharmingen), FITC-Gr1 (eBioscience), APC-F4/80 (BioLegend), FITC-CD43 (BD Pharmingen), APC-B220 (eBioscience), and PE-IgD (eBioscience).

### 5TGM1-GFP cell culture

The 5TGM1-GFP (5TGM1) cell line was originally obtained from Gregory Mundy at Vanderbilt University. *Samsn1* cDNA was subconed into an MSCV-PGK-Puro plasmid. MSCV-PGK-Puro plasmid and lentiviral vectors pCMVΔ8.9 and pM2G were transfected into HEK293T cells. Cell supernatant containing lentivirus was then plated on 5TGM1 cells and selected with puromycin for 72 hours. Cells were maintained in DMEM, 10% FBS, 1% penicillin-streptomycin. Proliferation was measured by BrdU ELISA (Roche Diagnostics).

### Immunoblotting

Antibodies: SAMSN1 (Sigma-Aldrich), actin (Sigma-Aldrich), and GFP (Santa Cruz Biotechnology). Blots were incubated with horseradish conjugated secondary antibodies (GE Healthcare) and visualized by chemiluminescence (Pierce Biotechnology).

### 5TGM1 *in vivo* tumors

1x10^6^ 5TGM1 cells were injected subcutaneously into the right flank of NOD-scid-IL2Rγ female mice. 14 days following tumor inoculation, 0.8x10^6^ ex vivo M2 polarized macrophages were injected directly into the tumor. Tumor volume was monitored by bidirectional precision caliper measurements (1/2 x length x width^2^). Maximum tumor volume was approximately 2cm^3^.

### Gene expression microarrays

RNA was extracted from C57BL/6 and KaLwRij BMMs (3 mice/strain) and BMSCs (2 mice/strain) using RNeasy Mini kit (Qiagen). RNA samples were submitted to the Genome Technology Access Center at Washington University School of Medicine for hybridization using the GeneChip Mouse Gene 1.0 ST array (Affymetrix). Differentially expressed genes were defined as ≥1.5 fold changed between C57BL/6 and KaLwRij.

We re-analyzed the microarray data of CD138+ plasma cells from healthy donors, MGUS patients, and MM patients published by Fonseca, R. et al. (GSE6477) [20, 21].

Data was analyzed using Partek Genomics Suite (Partek Inc.).

### Quantitative reverse transcription PCR

RNA was extracted using RNeasy Mini kit (Qiagen) and cDNA generated using iScript (Bio-Rad). Quantitative PCR was completed using SsoFast EVA Green Supermix or iQ Supermix (Bio-Rad). All samples run with biological replicates of ≥ 2. Primer sequences are in S1 Methods.

### Statistics

Data are shown as mean +/- SEM. Unless otherwise indicated, experiments were analyzed using Student’s t-test to compare 2 groups or ANOVA to compare multiple groups. *p<0.05; **p<0.01; ***p<0.001.

## Supporting Information

S1 Fig Immune responses are significantly different among mouse strains. Schema for immunization and serial serum sample protocol. Serum was collected at baseline (T0–5 months), post-primary immunization (T1–7 months), post-boosting immunization (T2–8.5 months), 12 months (T3), and 18 months (T4). Analysis of serial serum samples by ELISA for (b) immunoglobulin isotype G, (c) immunoglobulin isotype M, and (d) immunoglobulin isoype A. (e) Representative SPEP of mouse serum samples negative (-) and positive (+) for M-spike.
(PDF)

S2 Fig *Samsn1* is deleted in KaLwRij and expression varies by cell type in C57BL/6. (a) PCR amplification of the regions surrounding the 180kb deletion including *Samsn1* identified via WGS of the KaLwRij strain. Primers flanking the breakpoint amplified a product in KaLwRij genomic DNA and KaLwRij-derived 5TGM1 myeloma cell line DNA, but not C57BL/6 genomic DNA. (b) *Samsn1* mRNA expression was measured by RT-qPCR in multiple cell types. CD43- B cells were analyzed pre- and post-stimulation for 72hrs with IL4 and LPS. Macrophages were analyzed pre- and post-polarization to a M2 phenotype using IL4. CD45- BMSCs were also analyzed for *Samsn1* expression. ** P < 0.005, *** P < 0.0005.
(PDF)

S3 Fig *SAMSN1* is expressed at a lower level in human MGUS and MM plasma cells. Microarray data from CD138+ plasma cells from human healthy donors, MGUS patients, and MM patients, first published by R. Fonseca *et al*. in 2006 (GEO accession: GSE6477) was analyzed for *SAMSN1* expression levels. * *P < 0*.*05*, *** *P* > 0.0001.
(PDF)

S4 Fig FACS analysis of splenic B-cells. Isolated mouse splenocytes were negatively selected by magnetic immunodepletion, using anti-CD43 beads (Miltenyi Biotec). The negative fraction was then analysed by FACS. The CD43^−^ (middle panel) and naive B-cell (B220^+^, IgD^+^, right panel) populations are shown.
(PDF)

S5 Fig FACS analysis of bone marrow stromal cells. Whole bone marrow cells were cultured ascorbic acid-free αMEM, 10% FBS, 1% penicillin-streptomycin for 7 days in 5% oxygen (left panels, “pre-sorted”). On day 7, cells were negatively selected by MACS with anti-CD45 beads (right panels).
(PDF)

S6 Fig FACS analysis of bone marrow macrophages. Whole bone marrow was cultured in αMEM, 10% FBS, 1% penicillin-streptomycin, 50 ng/ml MCSF for 3 days. After 3 days, the cell population is enriched for GR1- / F4/80+ macrophages.
(PDF)

S7 Fig FACS analysis of bone marrow macrophages. Whole bone marrow was cultured in αMEM, 10% FBS, 1% penicillin-streptomycin, 50 ng/ml MCSF for 3 days. After 3 days, the cell population is enriched for GR1- / F4/80+ macrophages.
(PDF)

S1 Methods Supplemental section with expanded details regarding the experimental methods used in this manuscript. (DOCX)

S1 Table Mice with positive M-spike on serum protein electrophoresis. (XLSX)

S2 Table Candidate genes underlying genetic susceptibility to BIP in KaLwRij mice. (XLSX)

S3 Table Candidate genes underlying genetic susceptibility to multiple myeloma in humans. (XLSX)

S4 Table KaLwRij germline structural variants. (XLSX)

S5 Table KaLwRij germline SNVs. (XLSX)

S6 Table KaLwRij germline Indels. (XLSX)

S7 Table Frameshift and splice site mutations in the KaLwRij germline. (DOCX)

S8 Table Microarray analysis of BMM and BMSC gene expression. (XLSX)
