# Supplementary figures and images for: Whole Genome Sequence of Multiple Myeloma-Prone C57BL/KaLwRij Mouse Strain Suggests the Origin of Disease Involves Multiple Cell Types
Source: PLoS One. 2015 May 28;10(5):e0127828. doi: 10.1371/journal.pone.0127828 (PMC4447437; doi:10.1371/journal.pone.0127828)

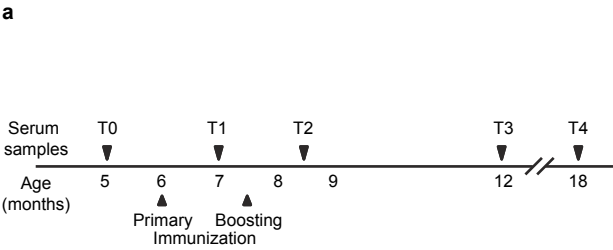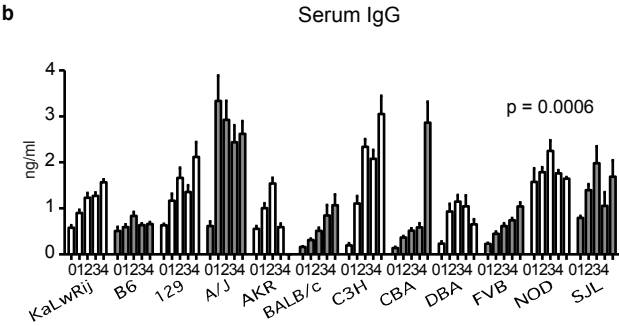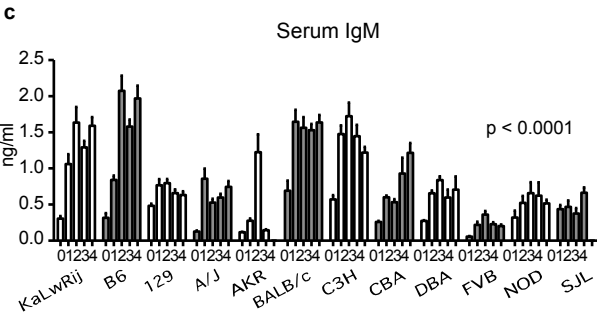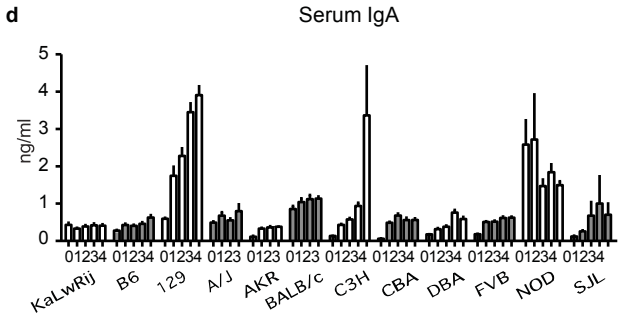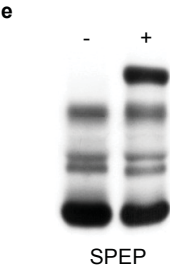

Supplement: S1 Fig — Schema for immunization and serial serum sample protocol. Serum was collected at baseline (T0–5 months), post-primary immunization (T1–7 months), post-boosting immunization (T2–8.5 months), 12 months (T3), and 18 months (T4). Analysis of serial serum samples by ELISA for (b) immunoglobulin isotype G, (c) immunoglobulin isotype M, and (d) immunoglobulin isoype A. (e) Representative SPEP of mouse serum samples negative (-) and positive (+) for M-spike. (PDF) [file pone.0127828.s001.pdf]

**a**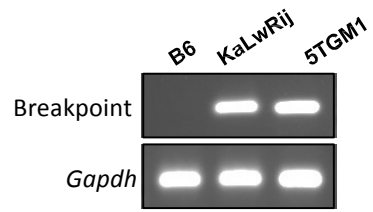**b**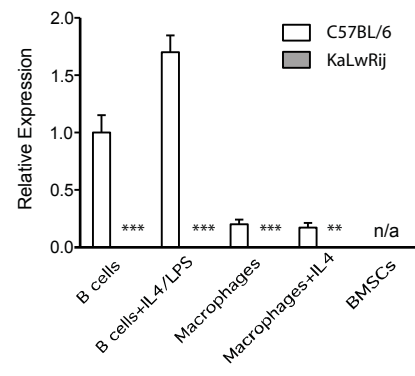

Supplement: S2 Fig — (a) PCR amplification of the regions surrounding the 180kb deletion including Samsn1 identified via WGS of the KaLwRij strain. Primers flanking the breakpoint amplified a product in KaLwRij genomic DNA and KaLwRij-derived 5TGM1 myeloma cell line DNA, but not C57BL/6 genomic DNA. (b) Samsn1 mRNA expression was measured by RT-qPCR in multiple cell types. CD43- B cells were analyzed pre- and post-stimulation for 72hrs with IL4 and LPS. Macrophages were analyzed pre- and post-polarization to a M2 phenotype using IL4. CD45- BMSCs were also analyzed for Samsn1 expression. ** P < 0.005, *** P < 0.0005. (PDF) [file pone.0127828.s002.pdf]

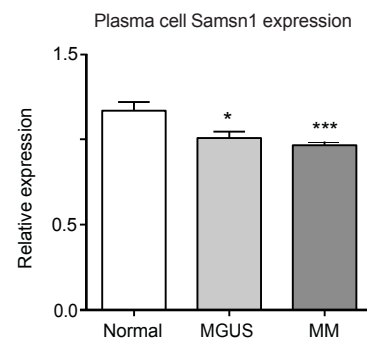

Supplement: S3 Fig — Microarray data from CD138+ plasma cells from human healthy donors, MGUS patients, and MM patients, first published by R. Fonseca et al. in 2006 (GEO accession: GSE6477) was analyzed for SAMSN1 expression levels. * P < 0.05, *** P > 0.0001. (PDF) [file pone.0127828.s003.pdf]

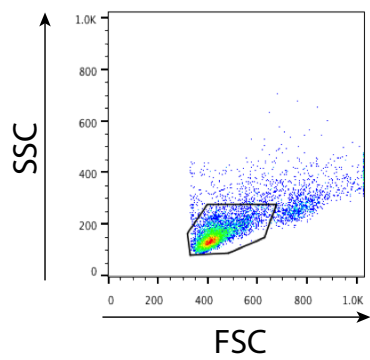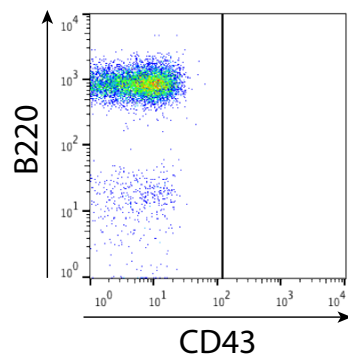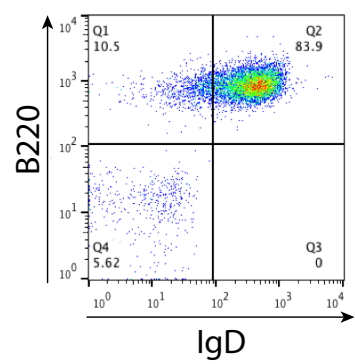

Supplement: S4 Fig — Isolated mouse splenocytes were negatively selected by magnetic immunodepletion, using anti-CD43 beads (Miltenyi Biotec). The negative fraction was then analysed by FACS. The CD43− (middle panel) and naive B-cell (B220+, IgD+, right panel) populations are shown. (PDF) [file pone.0127828.s004.pdf]

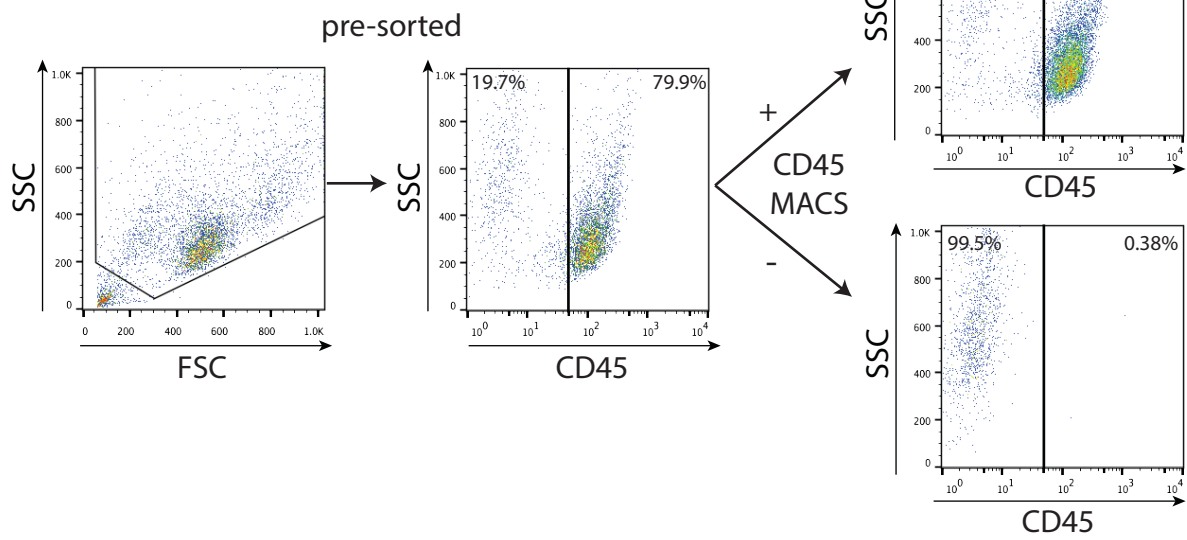

Supplement: S5 Fig — Whole bone marrow cells were cultured ascorbic acid-free αMEM, 10% FBS, 1% penicillin-streptomycin for 7 days in 5% oxygen (left panels, “pre-sorted”). On day 7, cells were negatively selected by MACS with anti-CD45 beads (right panels). (PDF) [file pone.0127828.s005.pdf]

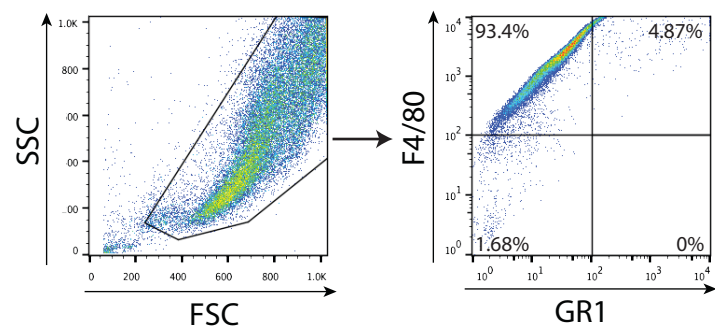

Supplement: S6 Fig — Whole bone marrow was cultured in αMEM, 10% FBS, 1% penicillin-streptomycin, 50 ng/ml MCSF for 3 days. After 3 days, the cell population is enriched for GR1- / F4/80+ macrophages. (PDF) [file pone.0127828.s006.pdf]
