# Supplementary material for: Whole Genome Sequence of Multiple Myeloma-Prone C57BL/KaLwRij Mouse Strain Suggests the Origin of Disease Involves Multiple Cell Types
Source: PLoS One. 2015 May 28;10(5):e0127828. doi: 10.1371/journal.pone.0127828 (PMC4447437; doi:10.1371/journal.pone.0127828)
Supplement: S7 Fig — Whole bone marrow was cultured in αMEM, 10% FBS, 1% penicillin-streptomycin, 50 ng/ml MCSF for 3 days. After 3 days, the cell population is enriched for GR1- / F4/80+ macrophages. (PDF) [file pone.0127828.s007.pdf]

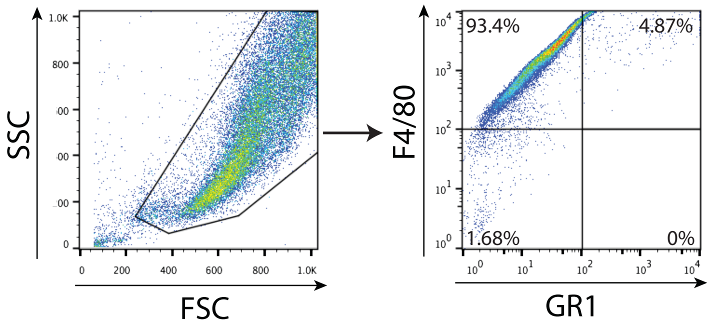

S7 Figure. FACS analysis of bone marrow macrophages. Whole bone marrow was cultured in αMEM, 10% FBS, 1% penicillin-streptomycin, 50 ng/ml MCSF for 3 days. After 3 days, the cell population is enriched for GR1- / F4/80+ macrophages.
