# Supplementary material for: Whole Genome Sequence of Multiple Myeloma-Prone C57BL/KaLwRij Mouse Strain Suggests the Origin of Disease Involves Multiple Cell Types
Source: PLoS One. 2015 May 28;10(5):e0127828. doi: 10.1371/journal.pone.0127828 (PMC4447437; doi:10.1371/journal.pone.0127828)
Supplement: S1 Methods — (DOCX) [file pone.0127828.s008.docx]

**Supplemental Information**

**METHODS**

**Immunization**

Mice were immunized with 50 µg chicken gamma globulin (Biosearch Technologies,) in complete or incomplete Freund’s adjuvant (Sigma-Aldrich) by intraperitoneal injection at 6 and 7.5 months of age. Serum immunoglobulin levels were monitored by ELISA (Bethyl Laboratories). Heritability (h2) was estimated as the ratio between strain variance to the total variance. SPEP was performed with the SPIFE 3000 analyzer and protein fractions quantified with the QuickScan 2000 densitometer (Helena Laboratories).

**Mouse phylogenetic analysis**

KaLwRij genomic DNA was isolated from kidney using DNeasy Blood & Tissue kit (Qiagen). SNP array analysis was performed using the Affymetrix mouse diversity genotyping array. Sample preparation, hybridization and scanning were performed by Jackson Laboratory according to the standard Affymetrix genotyping protocol. The PHYLIP package (University of Washington) was used for the phylogenetic analysis. A distance matrix was created by the Neighbor program based on the KaLwRij and the published SNP data of other 11 strains [[1](#_ENREF_1)]. The neighbor-joining algorithm was used to calculate a phylogenetic tree.

**C57BL/6 x KaLwRij mouse strain haplotype analysis**

SNP analysis was completed as previously described [[2](#_ENREF_2)]. KaLwRij SNPs were compared the published SNP data of C57BL/6 mice [[1](#_ENREF_1)]. All genes annotated in Ensembl mouse genome build 38 with ≥ 5 physically consecutive variants were included as candidate genes for further analysis.

**Genome-wide association study (GWAS)**

305 MM patients and 353 unaffected volunteers were genotyped using Affymetrix 6.0 platform. Data was analyzed by logistic regression analysis, adjusted for sex and group using PLINK (<http://pngu.mgh.harvard.edu/purcell/plink/)> [[3](#_ENREF_3)]. Genes containing SNPs in the 99th significance percentile were included as candidate genes. The study cohort was approved by the Human Research Protection Office at Washington University School of Medicine and at the Mayo Clinic. Informed consent from the patients was obtained in accordance with the Declaration of Helsinki.

**Whole genome sequencing of KaLwRij mouse**

Genomic DNA was isolated and pooled from splenocytes of six male KaLwRij littermates. Paired-end sequencing was performed on Illumina HiSeq2000 at the Genome Institute of Washington University in St. Louis. Sequences with base quality <13 at both read ends were trimmed and reads with less than 25 bp were removed. Reads were aligned to a reference genome (mm10) and each alignment was assigned a mapping quality score by the Burrows-Wheeler Aligner (BWA) [[4](#_ENREF_4)]. PCR duplicates were detected and removed (http://picard.sourceforge.net). After alignment, local realignment and basequality score recalibration were implemented in the GATK (<http://www.broadinstitute.org/gatk/index.php)> [[5](#_ENREF_5)].

SNPs and small insertions and deletions (INDEL) in the WGS sample were called by VarScan2 (http://varscan.sourceforge.net/) [[6](#_ENREF_6)]. Minimum coverage was set as 8X, minimum reads of variant allele as 3 and minimum proportion of variant allele as 30%. We used GATK for SNP and INDEL discovery across 4 exome samples simultaneously using variant quality score recalibration according to GATK Best Practices recommendations [[7](#_ENREF_7)]. Since the exomes came from the same genetic background, we only retained those germline variants that were presented in all 4 exome samples.

To identify structural variants (SVs), the aligned BAM from WGS was analyzed by clipping reveals structure (CREST) algorithm with default parameters [[8](#_ENREF_8)]. The predicted SVs were further filtered with >1 soft-clipped read on each side of breakpoint and with >5 total soft-clipped reads. For SNPs, we also used SIFT 4G annotator to predict whether an amino acid substitution affects protein function (http://sift-db.bii.a-star.edu.sg/) [[9](#_ENREF_9)].

The KaLwRij whole genome sequence is deposited in GenBank under accession number SRP057008.

To confirm Samsn1 deletion in KaLwRij, DNA was amplified using standard PCR and products were visualized on an agarose gel stained with ethidium bromide under UV light.

**Primer Sequences**

RT-qPCR (forward/reverse):

Cyclophilin: AGCATACAGGTCCTGGCATC / TTCACCTTCCCAAAGACCAC

Samsn1: TTCACGCCAAGTCCCTATGAC / TTCCCATTGGTGTTTTGCACATA

Chi3l3: GGGCATACCTTTATCCTGAG / CCACTGAAGTCATCCATGTC

Adipoq: CTCCACCCAAGGGAACTTGT / GGACCAAGAAGACCTGCATC

Fstl4: CTGCAGGTGAATGTGCCACC / TCTTGTGCCTGGGTTTCTGG

Tnfrsf22: ACCGGCTGTTCCTACTGTTAT / CGGAGGACAACAACGGACACA

Tnfrsf23: CTTCAACTGTCCCGATGGTGA / AGGCCATTATCTTTCCCTGTGA

Tnfrsf26: TTCAAACATGAGAACCTCTGCTG / CACATGGAGCACATTCACTCT

Gapdh: ACTTTGTCAAGCTCATTTCC / TGCAGCGAACTTTATTGATG

Ywhaz: ACTTTTGGTACATTGTGGCTTCAA / CCGCCAGGACAAACCAGTAT

Samsn1 Breakpoint Mapping (forward/reverse):

Gapdh: ACTTTGTCAAGCTCATTTCC / TGCAGCGAACTTTATTGATG

KaLwRij Samsn1 deletion: TTCAACTTAAAGGGCCAGCTA / CCTGGTGAGGAGGTGATGAT

**Supplemental Methods Legend**

Additional detailed methods and references not included in the main text are provided.

**IN-TEXT CITATIONS**

1. Didion JP, Yang H, Sheppard K, Fu CP, McMillan L, de Villena FP, et al. Discovery of novel variants in genotyping arrays improves genotype retention and reduces ascertainment bias. BMC genomics. 2012;13:34. doi: 10.1186/1471-2164-13-34. PubMed PMID: 22260749; PubMed Central PMCID: PMC3305361.

2. Burgess-Herbert SL, Cox A, Tsaih SW, Paigen B. Practical applications of the bioinformatics toolbox for narrowing quantitative trait loci. Genetics. 2008;180(4):2227-35. doi: 10.1534/genetics.108.090175. PubMed PMID: 18845850; PubMed Central PMCID: PMC2600954.

3. Purcell S, Neale B, Todd-Brown K, Thomas L, Ferreira MA, Bender D, et al. PLINK: a tool set for whole-genome association and population-based linkage analyses. American journal of human genetics. 2007;81(3):559-75. doi: 10.1086/519795. PubMed PMID: 17701901; PubMed Central PMCID: PMC1950838.

4. Chu L, Su MY, Maggi LB, Jr., Lu L, Mullins C, Crosby S, et al. Multiple myeloma-associated chromosomal translocation activates orphan snoRNA ACA11 to suppress oxidative stress. The Journal of clinical investigation. 2012;122(8):2793-806. doi: 10.1172/JCI63051. PubMed PMID: 22751105; PubMed Central PMCID: PMC3408744.

5. McKenna A, Hanna M, Banks E, Sivachenko A, Cibulskis K, Kernytsky A, et al. The Genome Analysis Toolkit: a MapReduce framework for analyzing next-generation DNA sequencing data. Genome research. 2010;20(9):1297-303. doi: 10.1101/gr.107524.110. PubMed PMID: 20644199; PubMed Central PMCID: PMC2928508.

6. Koboldt DC, Zhang Q, Larson DE, Shen D, McLellan MD, Lin L, et al. VarScan 2: somatic mutation and copy number alteration discovery in cancer by exome sequencing. Genome research. 2012;22(3):568-76. doi: 10.1101/gr.129684.111. PubMed PMID: 22300766; PubMed Central PMCID: PMC3290792.

7. Van der Auwera GA, Carneiro MO, Hartl C, Poplin R, Del Angel G, Levy-Moonshine A, et al. From FastQ data to high confidence variant calls: the Genome Analysis Toolkit best practices pipeline. Current protocols in bioinformatics / editoral board, Andreas D Baxevanis [et al]. 2013;11(1110):11 0 1- 0 33. doi: 10.1002/0471250953.bi1110s43. PubMed PMID: 25431634; PubMed Central PMCID: PMC4243306.

8. Wang J, Mullighan CG, Easton J, Roberts S, Heatley SL, Ma J, et al. CREST maps somatic structural variation in cancer genomes with base-pair resolution. Nature methods. 2011;8(8):652-4. doi: 10.1038/nmeth.1628. PubMed PMID: 21666668; PubMed Central PMCID: PMC3527068.

9. Kumar P, Henikoff S, Ng PC. Predicting the effects of coding non-synonymous variants on protein function using the SIFT algorithm. Nature protocols. 2009;4(7):1073-81. doi: 10.1038/nprot.2009.86. PubMed PMID: 19561590.
