# Supplementary material for: Whole Genome Sequence of Multiple Myeloma-Prone C57BL/KaLwRij Mouse Strain Suggests the Origin of Disease Involves Multiple Cell Types
Source: PLoS One. 2015 May 28;10(5):e0127828. doi: 10.1371/journal.pone.0127828 (PMC4447437; doi:10.1371/journal.pone.0127828)
Supplement: S7 Table — (DOCX) [file pone.0127828.s015.docx]

### S7 Table. Frameshift and splice site mutations in the KaLwRij germline.

| Chr | Start (bp) | End (bp) | Ref | Var | Alteration | Gene ID | Gene Name | Residue |
| --- | --- | --- | --- | --- | --- | --- | --- | --- |
| 4 | 108780610 | 108780619 | AGCGCGGCCG | - | Deletion | *3110021N24Rik* | RIKEN cDNA 3110021N24 gene | p.K58fs |
| 4 | 118893259 | 118893259 | C | - | Deletion | *Olfr1330* | Olfactory receptor 1330 | p.H59fs |
| 4 | 118916666 | 118916666 | T | - | Deletion | *Olfr1329* | Olfactory receptor 1329 | p.Y267fs |
| 4 | 147512870 | 147512870 | G | - | Deletion | *Gm13152* | Predicted gene 13152 | p.S228fs |
| 8 | 104182034 | 104182034 | C | - | Deletion | *Bean1* | Brain expressed, | p.L14fs |
|  |  |  |  |  |  |  | associated with Nedd4, 1 |  |
| 9 | 65280131 | 65280131 | G | - | Deletion | *Cilp* | Cartilage intermediate layer protein, nucleotide pyrophosphohydrolase | p.M1169fs |
| 11 | 3146255 | 3146256 | CG | - | Deletion | *Sfi1* | Sfi1 homolog, | p.R460fs |
|  |  |  |  |  |  |  | spindle assembly associated (yeast) |  |
| 11 | 69072547 | 69072547 | G | - | Deletion | *Tmem107* | Transmembrane protein 107 | p.M127fs |
| 3 | 93651613 | 93651613 | - | CT | Insertion | *Tdpoz2* | TD and POZ domain containing 2 | p.C351fs |
| 4 | 147512866 | 147512866 | - | C | Insertion | *Gm13152* | Predicted gene 13152 | p.Y227fs |
| 12 | 10370094 | 10370094 | - | AGTG | Insertion | *Nt5c1b* | 5'-nucleotidase, cytosolic IB | p.K11fs |
| 13 | 21484709 | 21484709 | - | A | Insertion | *Zkscan4* | Zinc finger with KRAB and SCAN domains 4 | p.G472fs |
| 16 | 32753370 | 32753370 | - | C | Insertion | *Muc4* | Mucin 4 | p.T1083fs |
| 11 | 55005842 | 55005842 | - | TGGA | Splicing | *Anxa6* | Annexin A6 | Exon 8 |
|  |  |  |  |  |  |  |  | Acceptor site |
| 12 | 105658157 | 105658157 | T | - | Splicing | *Atg2b* | Autophagy related 2B | Exon 14 |
|  |  |  |  |  |  |  |  | Acceptor site |
